# Study on Extraction and Antioxidant Activity of Flavonoids from *Hemerocallis fulva* (Daylily) Leaves

**DOI:** 10.3390/molecules27092916

**Published:** 2022-05-03

**Authors:** Wei Wang, Xiaoli Zhang, Qinglei Liu, Yucheng Lin, Zhiguo Zhang, Shanshan Li

**Affiliations:** 1 Engineering Research Center of Perfume & Aroma and Cosmetics of Ministry of Education, School of Perfume and Aroma Technology, Shanghai Institute of Technology, Shanghai 201418, China; wangweittg@sit.edu.cn (W.W.); zhangxiaolichn@163.com (X.Z.); liuqinglei@sit.edu.cn (Q.L.); phildoris@163.com (Y.L.); 2 School of Ecological Technology and Engineering, Shanghai Institute of Technology, Shanghai 201418, China; zgzhang@sit.edu.cn

**Keywords:** *Hemerocallis fulva* leaves, response surface methodology, flavonoids, antioxidant activity, in cellulo, in vitro

## Abstract

*Hemerocallis fulva* is a medical and edible plant. In this study, we optimized the ultrasound-assisted extraction (UAE) process of extracting flavonoids from *Hemerocallis fulva* leaves by single-factor experiments and response surface methodology (RSM). The optimum extraction conditions generating the maximal total flavonoids content was as follows: 70.6% ethanol concentration; 43.9:1 mL/g solvent to sample ratio; 61.7 °C extraction temperature. Under the optimized extraction conditions, the total flavonoid content (TFC) in eight *Hemerocallis fulva* varieties were determined, and *H. fulva* (L.) L. var. *kwanso* Regel had the highest TFC. The cytotoxicity of the extract was studied using the Cell Counting Kit-8 (CCK-8 assay). When the concentration was less than 1.25 mg/mL, the extract had no significant cytotoxicity to HaCaT cells. The antioxidant activity was measured via chemical antioxidant activity methods in vitro and via cellular antioxidant activity methods. The results indicated that the extract had a strong ABTS and •OH radical scavenging activity. Additionally, the extract had an excellent protective effect against H_2_O_2_-induced oxidative damage at a concentration of 1.25 mg/mL, which could effectively reduce the level of ROS to 106.681 ± 9.733% (*p* < 0.001), compared with the 163.995 ± 6.308% of the H_2_O_2_ group. We identified five flavonoids in the extracts using high-performance liquid chromatography (HPLC). Infrared spectroscopy indicated that the extract contained the structure of flavonoids. The results showed that the extract of *Hemerocallis fulva* leaves had excellent biocompatibility and antioxidant activity, and could be used as a cheap and potential source of antioxidants in the food, cosmetics, and medicine industries.

## 1. Introduction

*Hemerocallis fulva* also known as daylily and golden needle, is a perennial herb of Hemerocallis in Liliaceae [1]. *Hemerocallis fulva* has been widely planted in eastern Asia including in China, South Korea, and Japan [2]. It is a traditional Chinese plant that has been recorded in ancient books as being an edible and medicinal crop for thousands of years [3]. *Hemerocallis fulva* has been used as a conventional food in East Asia, with antivomiting, anti-inflammatory, diuretic, antidepressant, and sedation properties [2]. Some studies have shown that it has the effect of promoting sleep, and also is commonly used as an anti-inflammatory, for the treatment of skin burns [4]. Therefore, *Hemerocallis fulva* has attracted increasing attention from researchers because of its application potential in healthcare products and medicine [5]. *Hemerocallis fulva* is also a cheap source of bioactive substances as the leaves are a by-product of its cultivation [6]. A large number of leaves can be obtained during the necessary pruning of *Hemerocallis fulva* at maturity. The study indicated that flavonoids widely exist in *Hemerocallis fulva*, and they are one of the active components of *Hemerocallis fulva* [7]. Additionally, flavonoids have the functions of preventing and treating cardiovascular and cerebrovascular diseases, relieving cough, inhibiting bacteria, protecting the liver, and scavenging free radicals as antioxidants [8]. Therefore, it is important to study the content of total flavonoids in *Hemerocallis fulva* leaves. Oxidative stress is an imbalance between oxidation and antioxidation, which tends to increase oxidization and produce a large number of oxidative intermediates [9]. Excessive production of reactive oxygen species (ROS) can cause tissue damage and changes in cell function [10]. Oxidative stress is considered to be an important factor leading to disease and aging, which is due to the negative effect of free radicals in the body [11]. An increase in ROS has been associated with many diseases, including neurodegenerative diseases, cardiovascular diseases, and diabetes mellitus [12,13]. Natural antioxidants have attracted increasing attention since synthetic antioxidants may impart cytotoxicity and side effects on the body [14]. The main sources of natural antioxidants are herbs, fruits, vegetables, grains, and green and black tea [13,15]. Due to their potential anti-oxidation effect, extracting flavonoids from natural products is a major research focus in natural pharmaceutical chemistry [16].

ABTS and •OH assays are commonly used to determine antioxidant activity in vitro. Deseo et al. found that sugarcane molasses extract has excellent antioxidant activity, as determined by an ABTS assay [17]. Zhou et al. reported that the scavenging ability of yam polysaccharide against hydroxyl radicals reached the same level as ascorbic acid using an •OH assay [18]. Souto et al. used HaCaT (Human immortal keratinocyte) cells to evaluate the antioxidant activity and cytotoxicity of sustained-release drugs [19]. The ROS method is usually used to test the antioxidant activity at the cellular level [20]. Acero et al. detected changes in ROS concentration in the HepG2 cell line treated with cherry extracts using the ROS method [21].

In order to achieve optimal extraction conditions, we investigated the ultrasound-assisted extraction (UAE) process and explored the effects of ethanol concentration, solvent to sample ratio, and extraction temperature on the total flavonoid content (TFC) [22]. The TFC in leaves of eight *Hemerocallis fulva* varieties was analyzed to find the variety with the most abundant total flavonoid content. Furthermore, the antioxidant activity of extracts was studied by ABTS and •OH assays in vitro, and its cell-level antioxidant activity was investigated by the ROS method. Finally, the components of flavonoids in *Hemerocallis fulva* leaves were detected by HPLC. This study hoped to provide a basis for the large-scale industrial application of *Hemerocallis fulva* leaves as antioxidants.

## 2. Results and Discussion

### 2.1. Single-Factor Experiments

In the preliminary condition–optimization experiment, several important factors (ethanol concentration, solvent to sample ratio, extraction temperature, ultrasonic power, and extraction time) affecting the extraction of flavonoids were studied and analyzed.

#### 2.1.1. The Effect of Ethanol Concentration

**Figure 1:**
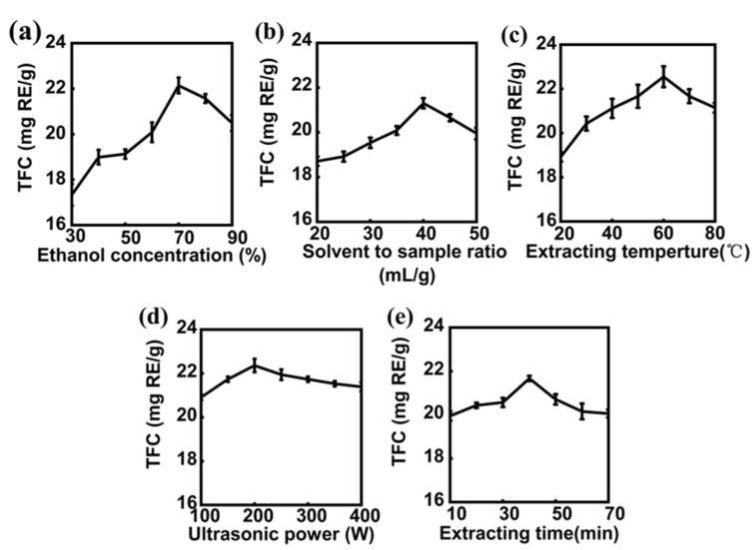
The effect of each single factor on the TFC of *Hemerocallis fulva* leaves extracts. (**a**) Ethanol concentration, (**b**) solvent to sample ratio, (**c**) extraction temperature, (**d**) ultrasonic power, and (**e**) extraction time. The data are shown as mean ± SD (*n* = 3), TFC: total flavonoid content.

Generally, methanol, ethanol, and acetone are suitable for extracting flavonoids [23]. Considering its safety and green extraction, ethanol was selected as the solvent [24]. In the single-factor experiments, we first studied the effect of ethanol concentration (30–90%) on the TFC in the extracts (Figure 1a). Aside from ethanol concentration, other conditions were set as follows: extraction temperature (50 °C), ultrasonic power (200 W), extraction time (40 min), and solvent to sample ratio (40:1 mL/g). At low concentration, the TFC maintained a stable increase with increasing ethanol concentration (30–70%). The TFC decreased when the ethanol concentration exceeded 70%. The extraction rate of other substances’ extracts other than flavonoids also increased, which affected the extraction of flavonoids and decreased the total flavonoids extracted from *Hemerocallis fulva* leaves [25,26]. Therefore, ethanol concentration (60–80%) was selected for the following RSM experiment.

#### 2.1.2. The Effect of Solvent to Sample Ratio

The effect of solvent to sample ratio (20:1–50:1 mL/g) on the TFC of extracts is shown in Figure 1b. Aside from this, other conditions were set as follows: ethanol concentration (70%), extraction temperature (50 °C), ultrasonic power (200 W), and extraction time (40 min). When the solvent to sample ratio was 20:1–40:1 mL/g, the TFC increased as the ratio increased. A higher ratio might accelerate mass transfer in the extraction process and promote the diffusion of more flavonoids into the solvent medium [23]. However, with a further increase in the ratio, it was observed that the TFC decreased slightly. The existence of too much solvent in the system might reduce the cavitation phenomenon due to there being lower nucleation sites, which might have a negative impact on the TFC [27]. This indicates that too low or too high a solvent to sample ratio was not conducive to the movement of flavonoids in plants towards the solvent under ultrasonic treatment [23]. When the ratio was 35:1–45:1 mL/g, the TFC was close to its peak. Therefore, based on the principle of reducing the amount of solvent and saving production costs, solvent to sample ratio (35:1–45:1 mL/g) was investigated for further RSM experiments.

#### 2.1.3. The Effect of Extraction Temperature

As shown in Figure 1c, the effects of extraction temperatures (20–80 °C) on the TFC were studied. KQ-250DB numerical control ultrasonic instrument was used in the UAE process. Its numerical control displayed the current temperature, and extraction temperature could be set. When the extraction temperature was reached, heating would be stopped automatically, thus avoiding the automatic increase in water temperature by using ultrasonic power for a long time [28]. Except for extraction temperatures, other conditions were set as follows: ultrasonic power (200 W), extraction time (40 min), ethanol concentration (70%), and solvent to sample ratio (40:1 mL/g). By increasing the extraction temperature from 20 °C to 60 °C, the content of total flavonoids increased significantly. This was because the higher extraction temperature accelerated the molecular movement, penetration, dissolution, and diffusion, which benefitted the release of flavonoids [23]. The TFC reached a peak when the extraction temperature was 60 °C, and the TFC decreased with the further increase in extraction temperature. Due to thermal instability, flavonoids might be oxidized at higher temperatures [29]. Therefore, a variable temperature range of 50–70 °C was selected to be used in the RSM experiment.

#### 2.1.4. The Effect of Ultrasonic Power

As shown in Figure 1d, the effects of ultrasonic power (100–400 W) on the TFC were studied. Aside from ultrasonic power, other conditions were set as follows: extraction time (40 min), ethanol concentration (70%), solvent to sample ratio (40:1 mL/g), and extraction temperature (50 °C). When the ultrasonic power was 100–200 W, the content of total flavonoids increased significantly with the increase in ultrasonic power. In a certain ultrasonic power range, the increase in ultrasonic power could produce a large number of cavitation bubbles and improve the transfer of flavonoids in the extracts to the solvent. [30]. When the ultrasonic power was 200 W, the content of total flavonoids reached its maximum and then decreased slowly. Excessive ultrasonic treatment might destroy the spatial structure of flavonoids [31]. Therefore, the ultrasonic power was selected to be 200 W in the RSM experiment.

#### 2.1.5. The Effect of Extraction Time

As shown in Figure 1e, the effects of extraction time (10–80 min) on the TFC were studied. Aside from extraction time, other conditions were set as follows: ethanol concentration (70%), solvent to sample ratio (40:1 mL/g), extraction temperature (50 °C), and ultrasonic power (200 W). At about 10–40 min, the TFC increased with the extension of extraction time. After extraction for 40 min, the content of total flavonoids reached its maximum and then showed a downward trend. Short-term ultrasonic treatment could promote the dissolution of flavonoids, while long-term ultrasonic treatment might destroy their structure [31,32]. Therefore, the extraction time was set to 40 min in the RSM experiment.

### 2.2. Optimization of UAE of Total Flavonoids from Hemerocallis fulva Leaves

#### 2.2.1. Effect of Extraction Variables on Total Flavonoids

Based on the results of single-factor experiment, three factors were selected for further RSM optimization using the Box–Behnken design (BBD) [33]. All variables were set at three levels, represented by −1, 0, and 1, respectively. As shown in Table 1, three factors were selected as variables to optimize the UAE, and the model was established and statistically analyzed in Table 1 and Table 2.

As shown in Table 3, multiple regression fitting was adopted on the experimental data to obtain the relationships between response variables. The yield of total flavonoids (Y_TFC_) was obtained by the conditions listed in Table 1 and Table 2, expressed in the form of polynomial equation. The quadratic regression equation was as follows:

Y_TFC_ = 23.01 − 0.35X_1_ + 1.00X_2_ + 0.19X_3_ − 0.69X_1_X_2_ + 0.15X_1_X_3_ − 0.41X_2_X_3_ − 1.49X_1_^2^ − 0.94X_2_^2^ − 0.59X_3_^2^ (1)

Analysis of variables was performed as shown in Table 3. The F value of multiple regression equation models was 82.43 (*p* < 0.0001), indicating that the change in response value caused by the change in independent variable was extremely significant in this model. The lack of fit was 0.1441 (*p* > 0.05), indicating that the model had a high degree of fitting. By ANOVA analysis of response surface regression parameters, R^2^ was 0.9907, which proved that there was a significant multiple regression relationship between the dependent variable and the selected independent variable, and 99.07% of the change in response value came from the selected variable.

According to the analysis of variance of the response value of the multiple regression equation, the primary terms (X_1_, X_2_, and X_3_) of the model were significantly different; the interactive terms (X_1_X_2_ and X_2_X_3_) were also significantly different. The difference in quadratic terms (X_1_^2^, X_2_^2^, and X_3_^2^) was very significant. This shows that the response surface and independent variables did not have a simple linear relationship.

#### 2.2.2. Response Surface Analysis

**Figure 2:**
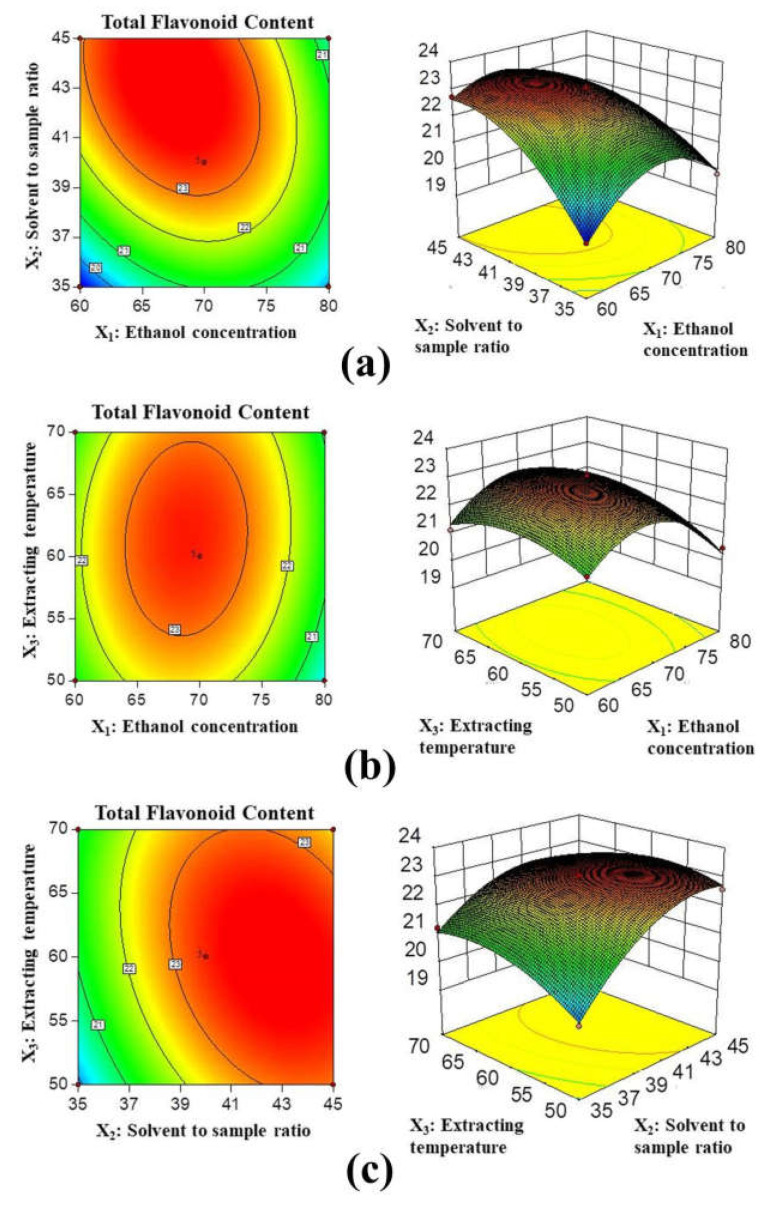
Contour plots and response surface plots of *Hemerocallis fulva* leaves extracts affected by ethanol concentration (X_1_), solvent to sample ratio (X_2_), and extraction temperature (X_3_) on the TFC. (**a**) ethanol concentration and solvent to sample ratio; (**b**) ethanol concentration and extraction temperature; (**c**) solvent to sample ratio and extraction temperature.

In the contour map, the values on a curve are the same. When they are oval-shaped or closely arranged, it indicates that the two factors have a greater impact on the response value [34]. In the response surface, the horizontal changes of the two factors have a great influence on the response value when the slope is steep [35]. From Figure 2a, it can be seen that the response surface is steep, and the changes in ethanol concentration (X_1_) and solvent to sample ratio (X_2_) have a great impact on the response value (*p* < 0.05). As seen from Figure 2b, the response surface of ethanol concentration (X_1_) and extraction temperature (X_3_) is relatively flat. The interaction effect of the two factors is not significantly different (*p* > 0.05). It can be seen from Figure 2c that the changes in the solvent to sample ratio (X_2_) and extraction temperature (X_3_) have a great impact on the response value. When the ethanol concentration is at the optimal value, the interaction curve of solvent to sample ratio (X_2_) and extraction temperature (X_3_) is an oval, indicating that the interaction between them is significant, and its *p*-value is 0.0040 (*p* < 0.05).

Based on the RSM method, the optimal conditions for extraction from *Hemerocallis fulva* leaves were as follows: the ethanol concentration was set at 70.6%, the solvent to sample ratio was set at 43.9:1 mL/g, and the extraction temperature was set at 61.7 °C. The experimental verification adopted the optimal extraction conditions, the theoretical value of TFC was 23.135 mg RE/g, and the experimental value was 23.621 ± 0.207 mg RE/g. This indicated that the model has a good fitting effect.

### 2.3. The TFC in Eight Hemerocallis fulva Varieties

**Figure 3:**
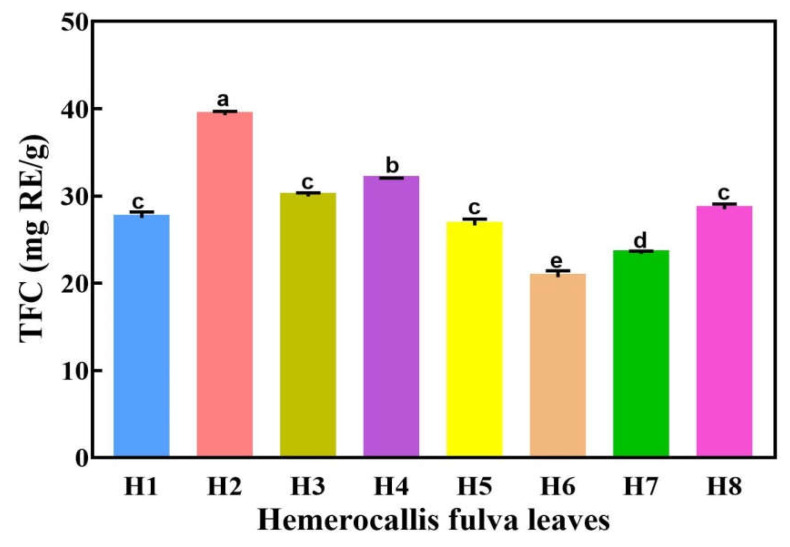
The TFC of eight *Hemerocallis fulva* varieties leaves. H1—*H.* “Gadsden Pinwheel”; H2—*H. fulva* (L.) L. var. *kwanso* Regel; H3—*H.* “Watermelon Slice”; H4—*H.* “Frans Hals”; H5—*H.* “Chicago Fire”; H6—*H.* “Stellar Double Rose”; H7—*H.* “Bela Lugosi”; H8—*H.* “Alexander Hay”. Different letters have significant differences in the mean at the 0.05 level.

The leaves of eight *Hemerocallis fulva* varieties were extracted by the best UAE process. As shown in Figure 3, the TFC of these leaves were determined. There was no significant difference in the TFC of Hemerocallis leaves among the four varieties (H1, H3, H5, and H8). Our results showed that the TFC of H2 was the highest, and could reach 39.26 ± 0.458 mg RE/g; the TFC in H6 was the lowest at 20.69 ± 0.763 mg RE/g. In the following section, H2 with the highest TFC was selected from the leaves of eight *Hemerocallis fulva* varieties to further explore its antioxidant activity and flavonoid components.

### 2.4. In Vitro Antioxidant Activity of Total Flavonoid Extracts from H2

**Figure 4:**
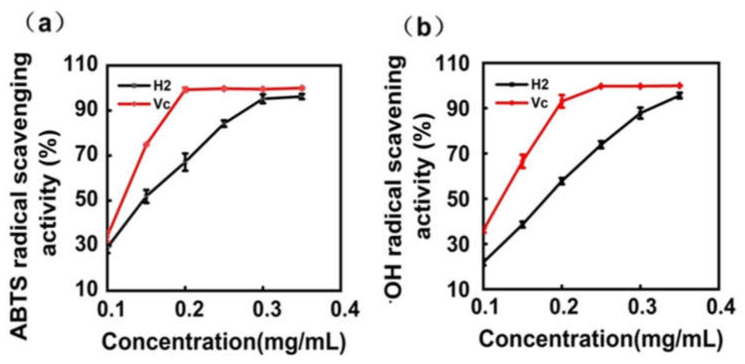
Antioxidant ability of the extract. (**a**) ABTS radical scavenging activity; (**b**) •OH radical scavenging activity. Data shown as the mean ± S.D. (*n* = 3). Vc: Ascorbic acid.

ABTS and •OH assays were adopted to determine the antioxidant activity of the extract, with ascorbic acid (Vc) as a control [36]. As shown in Figure 4, the extract was measured in the concentration range of 0.1 to 0.35 mg/mL, and antioxidant activity was concentration-dependent. When the concentration increased to 0.3 mg/mL, the antioxidant activity of the H2 extracts (93.83 ± 0.27%) was close to that of ascorbic acid (96.04 ± 2.20%) in the ABTS assay (Figure 4a). When the concentration of flavonoids increased to 0.35 mg/mL, the antioxidant activity of flavonoids (95.60 ± 1.23%) was close to that of ascorbic acid (99.92 ± 0.09%) in the •OH assay (Figure 4b). These results indicated that the extract had strong ABTS and •OH radical scavenging activity, and it was comparable to ascorbic acid.

### 2.5. Cellular Antioxidant Activity of Total Flavonoid Extracts from H2

#### 2.5.1. Effects of H2 and H_2_O_2_ on the Activity of HaCaT Cells

**Figure 5:**
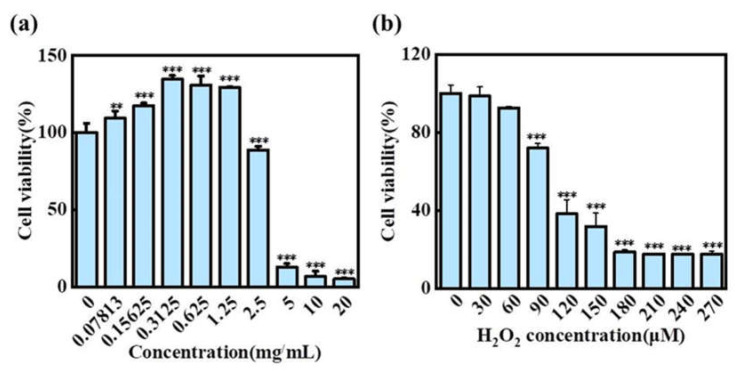
Effects of H2 and H_2_O_2_ on the activity of HaCaT Cells. (**a**) Cytotoxicity of the H2 extracts on HaCaT cells. (**b**) Cytotoxicity of different concentrations of H_2_O_2_ on HaCaT cells. Significance: *** *p* < 0.001, ** *p* < 0.01.

The CCK-8 method was used to explore the activity of the H2 extracts on HaCaT cells [37]. As shown in Figure 5a, the activity of HaCaT cells gradually increased with a concentration between 0.07813 and 1.25 mg/mL. When the concentration of the H2 extracts was lower than 2.5 mg/mL, HaCaT cell activity was higher than 90%. At the same time, when the concentration was lower than 1.25 mg/mL, HaCaT cell activity could be higher than 100%. This suggested that the low concentrations of the H2 extracts contribute to the growth of HaCaT cells. Therefore, we selected a H2 extract concentration of lower than 1.25 mg/mL to further explore the effect of the extract on ROS level. Many plant extracts have been proved to have antioxidant viability and to promote cell growth due to the presence of bioactive molecules produced in the process of plant growth, such as flavonoids, alkaloids, and polyphenols [38].

In order to determine the appropriate concentration for the H_2_O_2_ oxidative damage model, the responses of HaCaT cells to exposure to H_2_O_2_ (30–270 μM) were measured using a CCK-8 assay. As shown in Figure 5b, the cell viability decreased with an increase in H_2_O_2_ concentration, and a concentration of 90 μM resulted in about 50% cell mortality. As reported by Zhang et al., when the cell mortality rate was 50%, the cells were damaged and produced a large number of ROS [39]. Therefore, 90 μM H_2_O_2_ was selected as the optimal concentration for the cell oxidative damage model.

#### 2.5.2. Effect of H2 on ROS Level in HaCaT Cells Treated with H_2_O_2_

**Figure 6:**
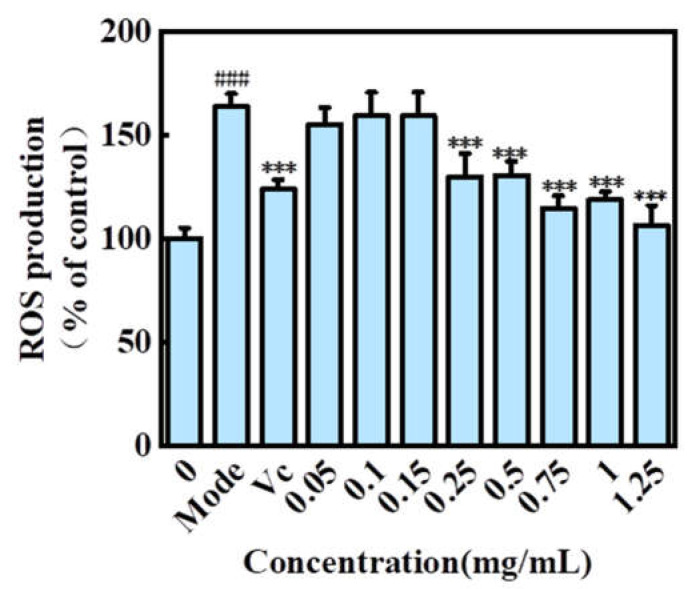
Effect of the H2 extracts on ROS level in HaCaT cells treated with H_2_O_2_. Mode is H_2_O_2_ group. Significance: ^###^ *p* < 0.001, *** *p* < 0.001.

As shown in Figure 6, the fluorescence intensity of H_2_O_2_ stimulated injury group was significantly higher than that of the control group (163.995 ± 6.308%, *p* < 0.001), indicating that H_2_O_2_ promoted the production of ROS in HaCaT cells. The ascorbic acid group (124.163 ± 4.601%, *p* < 0.001) could effectively retrain the production of ROS and protect HaCaT cells. The ROS level in HaCaT cells pretreated with H2 was lower than that in HaCaT cells pretreated with H_2_O_2_, which indicated that H2 could effectively reduce the ROS level in HaCaT cells induced by H_2_O_2_. H2 had good cell protection and prevented oxidative damage in the range of 0.25–1.25 mg/mL. These intracellular antioxidant properties might be related to the increase in endogenous antioxidant enzyme activity and the inhibition of ROS production [40].

### 2.6. HPLC Analysis

**Figure 7:**
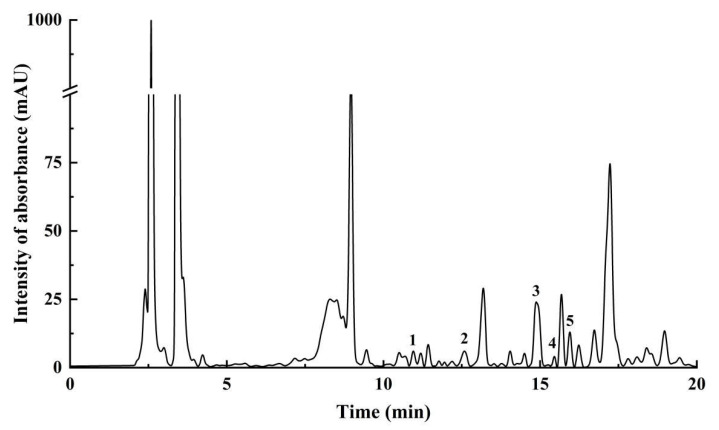
HPLC of the H2 extracts obtained by optimized UAE. 1—Catechin; 2—L-Epicatechin; 3—Rutin; 4—Hyperoside; 5—Isoquercitrin.

**Figure 8:**
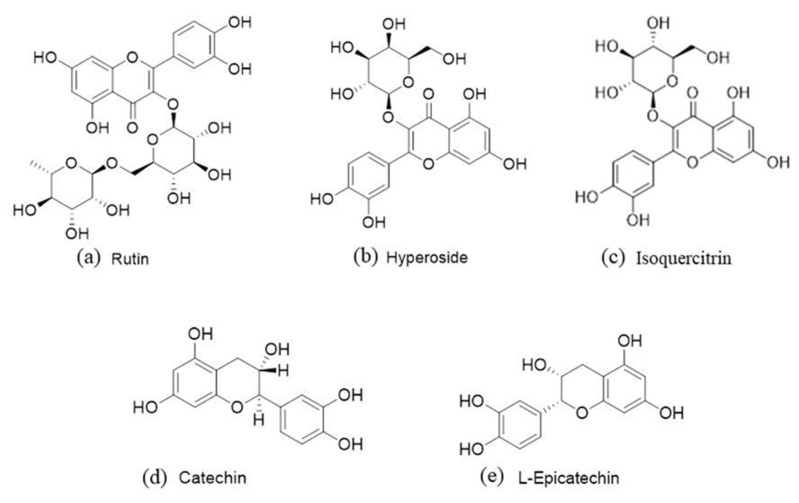
Chemical structures of flavonoids in the H2 extracts.

The flavonoids in the extracts were analyzed qualitatively and quantitatively using HPLC. Qualitative analysis was carried out by comparing the retention time of the standards and the extract [41,42]. The standards for HPLC analysis were selected according to the common flavonoids in the literature review, which have been reported in green leafy plants and *Hemerocallis fulva*. As shown in Figure 7, five kinds of flavonoids—including Rutin, Hyperoside, Isoquercitrin, Catechin, and L-Epicatechin—were detected to be consistent with the retention time of the standards. Hypericin and Catechin were beyond the quantitative range and their retention times were 15.501 min and 11.229 min, respectively. The contents of three quantifiable flavonoids at 254 nm are displayed in Table 4. The quantification of flavonoids in *Hemerocallis fulva* leaves was almost consistent with that reported by Szewczyk et al. [43]. The content of Rutin could reach 1001.316 ± 18.932 μg/g, close to that reported in the literature. The structural formula of flavonoids in the extracts is shown in Figure 8. Zhang et al. reported on lariciresinol, Roseoside, Phlomuroside, and other compounds in the methanol water extracts of Hemerocallis leaves. These compounds had strong antioxidant activity when the concentration was 50 μg/mL, which could exceed 72.7% [44].

### 2.7. Infrared Spectroscopy Analysis

**Figure 9:**
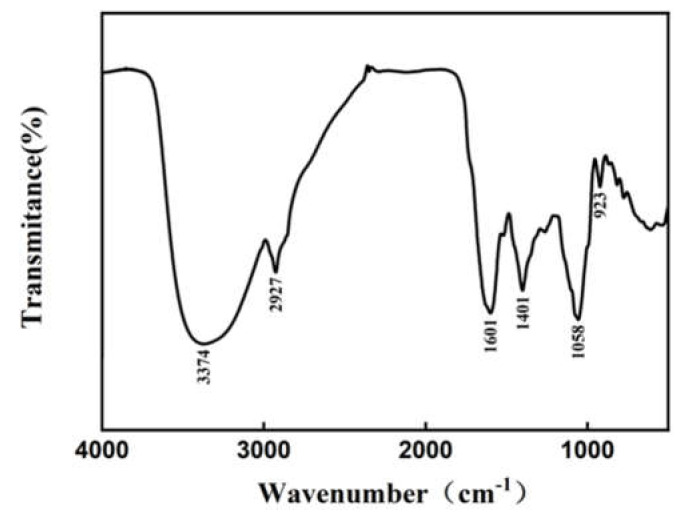
Infrared spectrum of the H2 extracts.

Infrared spectroscopy could be adopted to analyze the structure and chemical bonds of compounds and could be an effective ways to characterize and identify chemical compositions [45]. The infrared scanning spectrum of H2 extracts is shown in Figure 9. The sample had a strong absorption peak at 3374 cm^−1^, 1601 cm^−1^, and 1058 cm^−1^. There were strong and long absorption peaks at 3374 cm^−1^ and 1058 cm^−1^, which were attributed to the stretching vibration of O-H and C-O [46,47]. There was a weak absorption peak at 2927 cm^−1^, which might be the C-H stretching vibration peak of methyl [48]. In addition, the absorption peaks at 1401 cm^−1^ and 923 cm^−1^ were related to the bending vibration of C-H [49,50]. At 1601 cm^−1^, the strong absorption peak might be due to the C = C stretching vibration of aromatic compounds [51]. The diversity of functional groups indicated that there might be many kinds of bioactive substances such as flavonoids and phenols in the extracts, which further proved that the extract has good antioxidant activity.

## 3. Materials and Methods

### 3.1. Materials

The eight *Hemerocallis fulva* varieties (*H.* “Gadsden Pinwheel” (named biologist-Reinke, year of cultivation—1997)—H1; *H. fulva* (L.) L. var. *kwanso* Regel (Stout, 1917)—H2; *H.* “Watermelon Slice” (Scott-E., 1998)—H3; *H.* “Frans Hals” (Flory, 1955)—H4; *H.* “Chicago Fire” (Marsh, 1973)—H5; *H.* “Stellar Double Rose” (Brown-C., 1995)—H6; *H.* “Bela Lugosi” (Hanson-C., 1995)—H7; *H.* “Alexander Hay” (Holton, 2003)—H8) were introduced from the Netherlands and planted in the botanical garden of Shanghai Institute of Technology (30°50′ N, 121°30′ E, 6.67 m above sea level). *Hemerocallis fulva* leaves were collected from the botanical garden, washed, and dried in a vacuum freeze dryer at −50 °C. The dried *Hemerocallis fulva* leaves were crushed into powder with a pulverizer and passed through a 50-mesh sieve. Finally, they were put into sealed bags and stored at −20 °C in the laboratory until extraction.

### 3.2. Chemicals and Reagents

Ascorbic acid, 2,2′-azino-bis(3-ethylbenzothiazoline-6-sulfonic acid) (ABTS), phosphate buffer solution (PBS), ROS assay kit, CCK-8 kit, H_2_O_2_, and ethanol were purchased from Shanghai Titan Scientific Co., Ltd. (Shanghai, China). Fetal bovine serum and Dulbecco’s modified Eagle medium (DMEM) were obtained from Gibco (Carlsbad, CA, USA). Formic acid, acetonitrile, methanol, and water for HPLC analysis were from MACKLIN (Shanghai, China). All other chemicals were of analytical grade and were obtained from MACKLIN (Shanghai, China).

### 3.3. Ultrasound-Assisted Extraction (UAE)

The UAE method was selected for the extraction of *Hemerocallis fulva* leaves, referencing the method used by Pinto et al. with slight modification [52]. First, petroleum ether was used to remove grease from *Hemerocallis fulva* leaves [53]. Then, 20 g of *Hemerocallis fulva* leaf powder was accurately weighed and mixed with petroleum ether at a ratio of 1:10 g/mL with ultrasound for 15 min. Petroleum ether was removed by suction filtration. The obtained powder was mixed with ethanol and sonicated at a certain power and temperature. Then, the extract of *Hemerocallis fulva* leaves was obtained by centrifugation at 4 °C and 10,000 rpm for 10 min. After concentration by vacuum rotary evaporation, it was placed in vacuum for freeze-drying at −50 °C. Finally, the extract of lyophilized powder of *Hemerocallis fulva* leaves was obtained and stored in sealed bags at 4 °C until analysis.

### 3.4. Determination of Total Flavonoid Content (TFC)

The TFC was measured using the aluminum nitrate colorimetric method, as described by Cao et al. with modifications [54]. The extract (1 mL) and 5% NaNO_2_ solutions (1 mL) were mixed and reacted for 6 min. Then, 10% Al (NO_3_)_3_ solution (1 mL) was added and allowed to stand for 6 min. Finally, 4% NaOH solution (10 mL) was added and fully reacted. After 15 min, the absorbance was measured using an ultraviolet spectrophotometer at 510 nm [55]. The TFC was used as Rutin equivalent (RE)/g of the extract of *Hemerocallis fulva* leaves.

### 3.5. Selection of Variables

Multiple factors, such as solvent to sample ratio [56], extraction time [57], ethanol concentration [58], extraction temperature [59], and ultrasonic power [60] had significant effects on the TFC. Therefore, for the extraction of flavonoids from *Hemerocallis fulva* leaves, we selected ethanol concentration (30, 40, 50, 60, 70, 80, 90%), solvent to sample ratio (20, 25, 30, 35, 40, 45, 50 mL/g), extraction temperature (20, 30, 40, 50, 60, 70, 80 °C), ultrasonic power (100, 150, 200, 250, 300, 350, 400 W), and extraction time (10, 20, 30, 40, 50, 60, 70 min) as variables for single-factor experiments.

### 3.6. RSM Experiment Design

Based on single-factor experiment, the UAE extraction process of *Hemerocallis fulva* leaves was further optimized with the TFC. The three-factors-three-levels of the Box–Behnken design (BBD) was used to optimize the UAE extraction process [61]. Three factors (X_1_, X_2_, and X_3_) were used as independent variables and Y was used as response value. The fitting second-order polynomial regression model was as follows:

$$Y=\beta_{0}+\overset{k}{\underset{i=1}{\sum }}\beta_{i}X_{i}+\overset{k}{\underset{i=1}{\sum }}\beta_{ii\;}X_{i}^{2}+\overset{k-1}{\underset{i=1}{\sum }}\overset{k}{\underset{j>1}{\sum }}\beta_{ij}X_{i}X_{j}$$

where Y is TFC (mg RE/g), *X_i_* and *X_j_* are independent variables (*i* ≠ *j*), and *K* represents the number of test variables (*k* = 3). The regression coefficient was defined as the intercept ($\beta_{0}$), linear ($\beta_{i}$), quadratic ($\beta_{ii\;}$), and interaction terms ($\beta_{ij}$). Design Expert 10 was used for the analysis of variance (ANOVA) to determine the regression coefficients (*β*) of the model. The coefficient of determination (R^2^) was used to estimate the fitness of the polynomial equation. The significances of the dependent variables were statistically analyzed using the F-value and *p*-value (*p* < 0.05).

### 3.7. Antioxidant Assays

#### 3.7.1. Determination of ABTS Radical Scavenging Activity

ABTS radical scavenging activity was based on the method described by Cruz et al. with minor modifications [14]. First, 7-mM ABTS solution was mixed with 2.45-mM potassium persulfate in the same volume and reacted in the dark for 12–16 h. ABTS^•+^ stock solution was obtained by adjusting the absorbance value to 0.7 ± 0.02. The extract and ABTS^•+^ stock solution were thoroughly mixed and reacted at room temperature for 30 min under dark conditions. The absorbance was measured at 734 nm in a microplate reader [62].

#### 3.7.2. Determination of Hydroxyl (•OH) Radical Scavenging Activity

•OH radical scavenging activity was determined referring to the method described by Zhou et al. with slight modifications [63]. The extract (2 mL) was mixed with 9 mM salicylic acid (5 mL) and 9 mM ferrous sulfate (0.5 mL). Then, 8.8 mM H_2_O_2_ (0.5 mL) was added to the reaction, and the absorbance value was measured at 510 nm after 30 min.

### 3.8. Cellular Antioxidant Assay

#### 3.8.1. Cell Culture

HaCaT cells were acquired from the cell bank of the Chinese Academy of Sciences (Shanghai, China). DMEM included 10% fetal bovine serum and 1% antibiotic (100 U/mL of penicillin and 10 µg/mL of streptomycin). All cells were added to the DMEM and cultured in a carbon dioxide incubator (ESCO celmate) under a 5% CO_2_ atmosphere at 37 °C [64].

#### 3.8.2. Effects of H2 and H_2_O_2_ on the Activity of HaCaT Cells

The CCK-8 assay was adopted to measure the activity of the H2 extracts and H_2_O_2_ on HaCaT cells, as described by Zhang et al. [65]. HaCaT cells were cultured in 96-well plates at a density of 10,000 per well and placed in a carbon dioxide incubator at 37 °C and 5% CO_2_. After the cells adhered to the wall, the extract was added and cultured for 24 h. Finally, CCK-8 was added for 4 h to determine the absorbance value. The absorbance value was measured at 450 nm by a microplate reader. The percentage of HaCaT cell viability was calculated in the extracts and compared with the control group.

#### 3.8.3. Effects of H2 on the ROS Level in HaCaT Cells Treated with H_2_O_2_

ROS assay was used to determine the antioxidant activity of the H2 extracts on HaCaT cells, as described by Kostka et al. [66]. To explore the cellular antioxidant activity of the H2 extracts, the extract solution (20 mg/mL) was filtered by a 220 nm microporous membrane and diluted with the medium into the sample with concentrations of 0.07813–10 mg/mL. HaCaT cells were inoculated at a density of 1 × 10^4^ per well and were treated with the sample for 24 h before oxidative damage. Then, HaCaT cells were stimulated with the 90 µM concentration H_2_O_2_ for 2 h to construct a H_2_O_2_-induced oxidative damage model in vitro. The level of ROS in HaCaT cells was detected by the fluorescent probe DCFH-DA. After being cultured in the incubator for 20 min, the cells were washed with DMEM medium [67]. Finally, the fluorescence intensity was detected by a fluorescent enzyme labeling instrument. The emission wavelength was 488 nm, and the excitation wavelength was 525 nm.

### 3.9. HPLC Analysis

#### 3.9.1. Preparation of Standard Solution

We weighed 2.5 mg of Rutin, Hyperoside, Isoquercitrin, Catechin, and L-Epicatechin in a 25-mL brown volumetric flask, dissolved them with methanol, fixed the volume as standard stock solution and stored them in a refrigerator at 4 °C.

#### 3.9.2. HPLC Conditions

In this study, HPLC (Agilent, Santa Clara, CA, USA) was used for qualitative and quantitative analysis of the extract. The chromatographic column was Agilent Eclipse Plus C_18_ (4.6 mm × 250 mm, 5 μm). The mobile phase was an aqueous phase (A) (0.1% formic acid, V/V) and acetonitrile (B), and the flow rate was 1 mL/min. The column temperature was 35 °C. The gradient elution program was as follows: 0–2 min, 95–95% A; 2–10 min, 95%–80% A; 10–20 min, 80%–75% A; 20–25 min, 75%–75% A; 30–45 min, 75%–40% A. The detection wavelength of Rutin, Hyperoside, Isoquercitrin, Catechin, and L-Epicatechin was 254 nm with UV scanning.

### 3.10. Infrared Spectroscopy

The extract was mixed with potassium bromide at a ratio of 1:100, pressed into thin slices, and then analyzed with FTIR (Fourier Transform Infrared) Spectroscopy (Thermo Fisher Scientific, Waltham, MA, USA) [68]. The detection range of the infrared spectrum was 4000 cm^−1^ to 500 cm^−1^.

### 3.11. Statistical Analyses

Design Expert 10 was used to design the response surface experiment in order to optimize the extraction process. A *p*-value below 0.05 was considered statistically significant. All results were expressed in the form of mean ± standard deviation (SD) of three independent tests. Origin 2021, GraphPad Prism 7, and SPSS 25 were used for data processing and analysis.

## 4. Conclusions

In this paper, the ultrasound-assisted extraction method was successfully utilized for flavonoids from *Hemerocallis fulva* leaves with Box–Behnken design. In this work, the optimal extraction conditions were obtained by evaluating three factors and verified by the experimental value. H2 had the highest total flavone content (39.26 ± 0.458 mg RE/g) in the leaves of eight *Hemerocallis fulva* varieties. The extract indicated strong free radical scavenging ability against ABTS and •OH free radicals. At a concentration of 1.25 mg/mL, the extract had no cytotoxicity towards HaCaT cells and had a protective effect against H_2_O_2_-induced oxidative damage in the cells. HPLC was applied for qualitative and quantitative analysis of three flavonoids including Rutin, Isoquercitrin, and L-Epicatechin in the extracts. This study provides data-backed support regarding the efficiency of the extraction process, excellent antioxidant ability, and protective effect towards H_2_O_2_-injured cells of the flavonoids of *Hemerocallis fulva* leaves. Based on these results, *Hemerocallis fulva* leaves can provide potential and cheap raw materials for the development of natural antioxidants in drugs, cosmetics, and functional foods.

## Figures and Tables

**Table 1:** Level and code of factors chosen for the RSM experiment.

Level	Factor		
	Ethanol Concentration (X_1_) (%)	Solvent to Sample Ratio (X_2_) (mL/g)	Extraction Temperature (X_3_) (°C)
−1	60	35	50
0	70	40	60
1	80	45	70

**Table 2:** The RSM experiment design and results.

Runs	Ethanol Concentration (X_1_) (%)	Solvent to Sample Ratio (X_2_) (mL/g)	Extraction Temperature (X_3_) (°C)	TFC (mg RE/g)	
				Actual Value	Predicted Value
1	−1 (60)	0 (40)	1 (70)	21.12	21.33
2	0 (70)	−1 (35)	−1 (50)	19.78	19.89
3	−1 (60)	0 (40)	−1 (50)	21.29	21.25
4	0 (70)	0 (40)	0 (60)	23.12	23.01
5	0 (70)	0 (40)	0 (60)	23.05	23.01
6	1 (80)	−1 (35)	0 (60)	19.82	19.92
7	0 (70)	1 (45)	−1 (50)	22.56	22.71
8	1 (80)	1 (45)	0 (60)	20.50	20.56
9	1 (80)	0 (40)	1 (70)	20.88	20.93
10	−1 (60)	−1 (35)	0 (60)	19.30	19.24
11	0 (70)	1 (45)	1 (70)	22.37	22.27
12	−1 (60)	1 (45)	0 (60)	22.73	22.62
13	1 (80)	0 (40)	−1 (50)	20.47	20.26
14	0 (70)	0 (40)	0 (60)	23.12	23.01
15	0 (70)	0 (40)	0 (60)	22.98	23.01
16	0 (70)	−1 (35)	1 (70)	21.23	21.08
17	0 (70)	0 (40)	0 (60)	22.79	23.01

**Table 3:** ANOVA for the fitted quadratic polynomial model for UAE of TFC from *Hemerocallis fulva* leaves.

Source	Sum of Squares	df	Mean Square	F Value	*p*-Value (Prob > F)
Model	27.81	9	3.09	82.43	<0.0001 ***
X_1_	0.96	1	0.96	25.58	0.0015 **
X_2_	8.08	1	8.08	215.43	<0.0001 ***
X_3_	0.28	1	0.28	7.40	0.0297 *
X_1_X_2_	1.88	1	1.88	50.20	0.0002 ***
X_1_X_3_	0.086	1	0.086	2.30	0.1728
X_2_X_3_	0.67	1	0.67	17.76	0.0040 **
X_1_^2^	9.29	1	9.29	247.81	<0.0001 ***
X_2_^2^	3.72	1	3.72	99.17	<0.0001 ***
X_3_^2^	1.45	1	1.45	38.55	0.0004 ***
Residual	0.26	7	0.037		
lack of Fit	0.19	3	0.062	3.22	0.1441
Pure Error	0.077	4	0.019		
Cor Total	28.07	16			
SD	0.19				
Mean	21.6		R^2^	0.9907	
C.V.%	0.90		R^2^ adj	0.9786	

Cor. Total—corrected total; df—degree of freedom; R^2^—determination coefficient; R^2^ adj—adjusted R^2^; C.V.% —variation coefficient value; Significance—*** *p* < 0.001, ** *p* < 0.01, * *p* < 0.05.

**Table 4:** Composition of the H2 extracts obtained by optimized UAE.

Analyte	Molecular Formula	Equation	R^2^	RT (min)	Concentration (μg/g Dry Weight)
Rutin	C_27_H_30_O_16_	y = 20.98727x + 10.52446	0.9988	14.711	1001.316 ± 18.932
Isoquercitrin	C_21_H_20_O_12_	y = 22.82917x + 4.57751	0.9989	15.755	474.996 ± 5.768
L-Epicatechin	C_15_H_14_O_6_	y = 6.12946x − 11.33588	0.9931	12.515	423.814 ± 2.330

## Data Availability

The data presented in this study are available on request from the corresponding author.

## Sample Availability

Samples of *Hemerocallis fulva* leaves extracts are available from the authors.
